# The prostate-gland asymmetry affects the 3- and 12-month continence recovery after RARP in patients with small prostate glands: a single center study

**DOI:** 10.1007/s11701-024-02024-3

**Published:** 2024-08-09

**Authors:** Francesco Di Bello, Simone Morra, Agostino Fraia, Gabriele Pezone, Federico Polverino, Giuliano Granata, Claudia Collà Ruvolo, Luigi Napolitano, Andrea Ponsiglione, Arnaldo Stanzione, Roberto La Rocca, Raffaele Balsamo, Massimiliano Creta, Massimo Imbriaco, Ciro Imbimbo, Nicola Longo, Gianluigi Califano

**Affiliations:** 1 Department of Neurosciences, Reproductive Sciences and Odontostomatology, University of Naples “Federico II”, Via Sergio Pansini 5, 80131 Naples, Italy; 2 Department of Advanced Biomedical Sciences, University of Naples “Federico II”, 80131 Naples, Italy; 3 Urology Unit, AORN Ospedali dei Colli, Monaldi Hospital, 80131 Naples, Italy

**Keywords:** MRI, PSA, Prostate cancer, IPSS, Urinary incontinence

## Abstract

To test the impact of the prostate-gland asymmetry on continence rates, namely 3- and 12-month continence recovery, in prostate cancer (PCa) patients who underwent robot-assisted radical prostatectomy (RARP). Within our institutional database, RARP patients with complete preoperative MRI features and 12 months follow-up were enrolled (2021–2023). The population has been stratified according to the presence or absence of prostate-gland asymmetry (defined as the presence of median lobe or side lobe dominance). Multivariable logistic regression models (LRMs) predicting the continence rate at 3 and 12 months after RARP were fitted in the overall population. Subsequently, the LRMs were repeated in two subgroup analyses based on prostate size (≤ 40 vs > 40 ml). Overall, 248 consecutive RARP patients were included in the analyses. The rate of continence at 3 and 12 months was 69 and 72%, respectively. After multivariable LRM the bladder neck sparing approach (OR 3.15, 95% CI 1.68–6.09, *p* value < 0.001) and BMI (OR 0.90, 95% CI 0.82–0.97, *p* = 0.006) were independent predictors of recovery continence at 3 months. The prostate-gland asymmetry independently predicted lower continence rates at 3 (OR 0.33, 95% CI 0.13–0.83, *p* = *0.02*) and 12 months (OR 0.31, 95% CI 0.10–0.90, *p* = 0.03) in patients with prostate size ≤ 40 ml. The presence of prostate lobe asymmetry negatively affected the recovery of 3- and 12-months continence in prostate glands ≤ 40 mL. These observations should be considered in the preoperative planning and counseling of RARP patients.

## Introduction

Radical prostatectomy (RP) represents a treatment option for the prostate cancer (PCa) management, especially for patients with long life expectancy [1]. Over the past few decades, RP techniques have advanced to improve cancer control, without impairing pelvic organ function [2, 3]. Despite the minimally invasive approaches (such as robot-assisted RP [RARP]), one of the major concerns remains the post-operative urinary incontinence (UI) [4]. Indeed, the rates of UI after RARP remain relatively high, ranging from 30 to 50% within the first 3 months until 10% within 12 months, postoperatively [5], impairing the patients’ quality of life (QoL) [6–8].

Historically, several preoperative clinical and surgical predictors of UI after RARP have been described, such as the prostate volume (PV) or the bladder neck preservation [9, 10]. In the current scenario, the significance of multiparametric prostatic magnetic resonance imaging (mpMRI) is progressively gaining prominence [2, 11]. Nowadays, MRI is widely used to measure parameters of the pelvic floor structures that could play a pivotal role in post-operative continence recovery, such as membranous urethral length (MUL), prostate shape or surgical urethral length preservation [12–16]. Specifically, previous reports showed better continence recovery for prostate shape types D (defined as prostatic apex not overlapping the membranous urethra anteriorly and posteriorly according to Lee definition) than the other counterparts [13, 14].

To the best of our knowledge no previous authors investigated the impact of prostate-gland asymmetry (defined as the presence of median lobe or side lobe dominance) in predicting continence rates after RARP. We addressed this void. Specifically, the aim of the current study is to test the impact of the prostate-gland asymmetry on 3- and 12-months continence recovery after RARP.

## Materials and methods

The need for informed consent was waived by the institutional review board, which approved this retrospective study.

### Study population

We retrospectively identified PCa patients who underwent RARP at the Urologic Department of the University of Naples Federico II, between September 2021 and September 2023. Only patients that satisfied the follow inclusion criteria were included: (i) RARP performed within 6 months from mpMRI; (ii) mpMRI records; (iii) outpatient evaluation at 3 and 12 months after RARP. The exclusion criteria were: (i) mpMRI not retrieved or not accessible; (ii) missing data at follow-up. All PCa patients were naïve for previous benign prostatic surgery before RARP (only three patients underwent previous BPH surgery and were excluded for further analyses). The surgical procedure consisted of trans-peritoneal no-Retzius sparing approach with nerve sparing (NS) and bladder neck preservations (according to the oncologic features and prostate morphology). The dorsal venous complex is always sutured with a V-lock running suture. The lymph node dissection was performed in intermediate risk, according to the Briganti nomogram, and high-risk patients [17]. In each procedure, the reconstruction of the posterior plane, according to the Rocco approach, and a standard single-knot Van Velthoven vesico-urethral anastomosis were performed [18]. All patients underwent cystography before catheter removal between the fifth and seventh post-operative day. All RARP procedures were performed or tutored by surgeons with high expertise in robotic surgery (more than 300 procedures). Urinary and sexual rehabilitation started immediately after catheter removal by pelvic floor exercises and Tadalafil 20 mg (3/week) [19].

### Variable definition

For each patient included, the following clinical variables were recorded: age at RARP (continuously coded), body mass index (BMI, kg/m^2^), International Prostate Symptom Score (IPSS) score, Q max (categorized as: ≤ 12 vs > 12 mL/sec), post-void residual (PVR, categorized as: ≤ 50 vs > 50 mL), prostate volume (PV, categorized as: ≤ 40 vs > 40 mL), nerve sparing (NS, no vs yes), and bladder neck sparing (BNS; no vs yes). Moreover, the following mpMRI feature was recorded: prostate-gland asymmetry (defined as the presence of median lobe or side lobe dominance; no vs yes). Specifically, the presence of prostate lobe asymmetry was identified in the main three sections of mpMRI recording: sagittal, frontal and axial. Here, where the presence of prostate lobe asymmetry was declared as found if it was present in at least two sections of mpMRI recording. The mpMRI review was assessed by two investigators with high expertise in prostate mpMRI interpretation (a senior urology resident and an Assistant Professor). Subsequently, two senior radiologists and a radiology Ordinary Professor confirmed the results. The primary outcome analyzed was the rate of urinary continence defined as the use of no pad/day or one safety pad/day at 3- (early continence) and 12-months after RARP [4, 13].

### Statistical analysis

Descriptive statistics were presented as medians and interquartile ranges (IQR) for continuously coded variables or counts and percentages for categorically coded variables. The Wilcoxon rank sum test and Pearson’s Chi-square test examined the statistical significance of ‘medians’, and proportions’ differences. The results were tabulated according to the prostate lobe asymmetry (Group 1: no vs Group 2: yes). Two sets of analyses were in addition performed. First, in the overall population two independent univariable and multivariable logistic regression models (LRMs), adjusted for prostate-gland asymmetry, PV, age, BMI, IPSS, and BNS approach as well as NS approach, were fitted to predict the continence recovery rate at 3 and 12 months after RARP. Second, according to PV (≤ 40 mL and > 40 mL), two independent univariable and multivariable LRMs, adjusted for prostate-gland asymmetry, IPSS, and BNS approach, were fitted. In all statistical analyses, the R software environment for statistical computing and graphics (R version 3.6.1) was used. All tests were two-sided with a level of significance set at *p* < 0.05 (Tables 1, 2, 3).

**Table 1:** Clinical characteristic of 248 PCa patients, underwent RARP at our single-tertiary referral academic center between 2020 and 2023, stratified according to the prostate lobe asymmetry (defined as the presence of median lobe or side lobe dominance; no or yes)

	Overall, *N* = 248	Absence of prostate lobe asymmetry, *N* = 116 (47%)^1^	Presence of prostate lobe asymmetry, *N* = 132 (53%)^1^	*P* value^1^
Age (years at RARP), median (IQR)	67 (62, 72)	67 (62, 71)	67 (64, 72)	0.2
BMI (kg/m^2^), median (IQR)	26.0 (23.9, 28.5)	26.0 (23.9, 28.1)	26.2 (23.9, 28.7)	0.9
IPSS score, median (IQR)	8 (4, 15)	7 (3, 15)	10 (4, 15)	0.3
Prostate volume (mL), *n* (%)				**0.004**
≤ 40	90 (36%)	53 (46%)	37 (28%)	
> 40	158 (64%)	63 (54%)	95 (72%)	
*Q*_max_, *n* (%)				0.07
≤ 12 ml/sec	46 (19%)	27 (23%)	19 (14%)	
> 12 ml/sec	202 (81%)	89 (77%)	113 (86%)	
PVR, *n* (%)				0.7
≤ 50 ml	149 (60%)	71 (61%)	78 (59%)	
> 50 ml	99 (40%)	45 (39%)	54 (41%)	
Nerve sparing				0.08
No	66 (27%)	25 (22%)	41 (32%)	
Yes	179 (73%)	90 (78%)	89 (68%)	
Bladder neck sparing				0.08
No	130 (52%)	54 (47%)	76 (58%)	
Yes	118 (48%)	62 (53%)	56 (42%)	

*BMI*, body mass index; *IQR*, interquartile range; *IPSS*, International Prostate Symptom Score; *n*, number; *PCa*, prostate cancer; *PSAD*, prostate-specific antigen density; *PVR*, Post-void residual; *RARP*, robot-assisted radical prostatectomy

^1^Wilcoxon rank sum test; Pearson’s Chi-square test

**Table 2:** Multivariable logistic regression model predicting A) 3-mo continence recovery and B) 12-mo continence recovery in 248 PCa patients, underwent RARP at our single-tertiary referral academic center between 2021–2023

Characteristic	CONTINENCE			
	(A) 3-MONTHS		(B) 12-MONTHS	
	OR (95% CI)	*P* value	OR (95% CI)	*P* value
Prostate gland asymmetry				
No	Ref.	–	Ref.	–
Yes	1.01 (0.59–1.80)	0.9	1.10 (0.58–2.07)	0.7
Prostate volume				
≤ 40	Ref.	–	Ref.	–
> 40	0.75 (0.42–1.33)	0.3	0.97 (0.51–1.82)	0.8
Age	0.95 (0.91–1.00)	0.05	0.99 (0.94–1.04)	0.8
BMI	0.90 (0.82–0.97)	**0.006**	0.95 (0.87–1.04)	0.2
IPSS	0.97 (0.92–1.01)	0.1	9.96 (0.93–1.01)	0.1
Bladder neck sparing				
No	Ref.	–	Ref.	–
Yes	3.15 (1.68–6.08)	** < 0.001**	3.51 (1.87–6.67)	** < 0.001**
Nerve sparing				
No	Ref.	–	Ref.	–
Yes	1.56 (0.90–2.73)	0.1	0.88 (0.48–1.63)	0.7

Bold indicates the statstically significant results

*CI*, Confidence Interval; *IPSS*, International Prostate Symptom Score; *OR*, Odds Ratio; *PCa*, prostate cancer; RARP, robot-assisted radical prostatectomy

**Table 3:** *A*) Multivariable LRM predicting 3-mo continence and B) Multivariable LRM predicting 12-mo continence in: 90 RARP patients with a prostate volume ≤ 40 ml vs 158 RARP patients, with a prostate volume > 40 ml

	CONTINENCE							
	(A) 3-MONTHS				(B)12-MONTHS			
Prostate volume	≤ 40 ml		> 40		≤ 40 ml		> 40	
Characteristic	OR (95% CI)	*P* value	OR (95% CI)	*P* value	OR (95% CI)	*P* value	OR (95% CI)	*P* value
Prostate gland asymmetry								
No	Ref.	–	Ref.	–	Ref.	–	Ref.	–
Yes	0.33 (0.13–0.83)	**0.02**	1.65 (0.84, 3.28)	0.1	0.31 (0.10–0.90)	**0.03**	2.14 (1.00–4.60)	0.05
IPSS	0.93 (0.87–1.01)	0.06	0.96 (0.92–0.99)	**0.03**	0.91 (0.84–0.99)	**0.03**	0.98 (0.93–1.03)	0.4
Bladder neck sparing								
No	Ref.		–	Ref.	Ref.	–	Ref.	–
Yes	2.72 (0.96–8.11)	0.06	2.71 (1.50–5.04)	**0.001**	5.10 (1.67–16.73)	**0.005**	3.32 (1.52–7.33)	**0.003**

Bold indicates the statstically significant results

## Results

### Main study population

According to the inclusion criteria, 248 PCa patients who underwent RARP at our center were included. Of all, 116 (47%) and 132 (53%) RARP patients had no (group 1) and yes (group 2) prostate-gland asymmetry, respectively. According to PV, 46% of Group 1 and 28% of Group 2 patients had a prostate gland ≤ 40 mL, while 54% of Group 1 and 72% of Group 2 RARP patients had a prostate size > 40 mL (*p* = 0.004). No statistically significant differences were recorded between the two groups according to the IPSS scores, Q max, the PVR, the NS and the BNS approaches (all *p* > 0.05) (Table. 1).

**Fig. 1:**
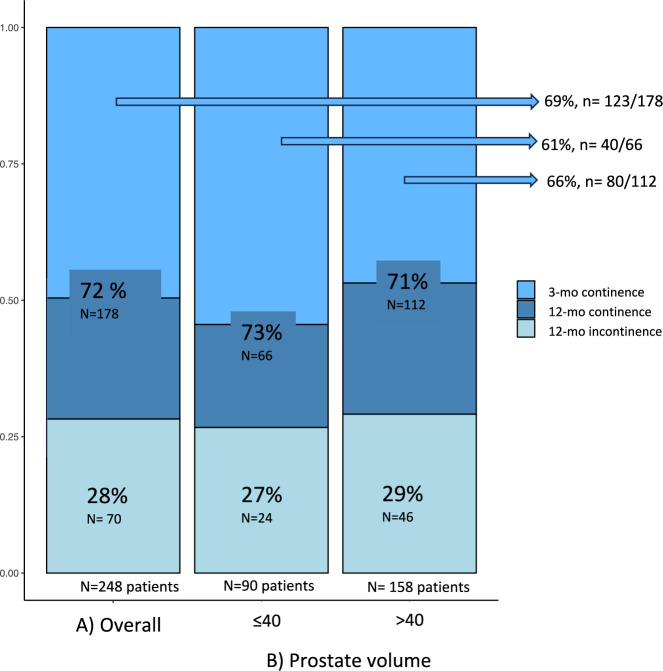
**A** The overall rates of 12-month continence and incontinence in 248 PCa patients, underwent RARP at our single-tertiary referral academic center between 2021–2023; **B** The rates of 3-month continence, 12-month continence, 12-month incontinence in 248 PCa patients, stratified according to the prostate volume (≤ 40 vs. > 40 ml)

### Multivariable logistic regression models predicting continence at 3 and 12 months after RARP in the overall population

In the overall population, the rates of continence at 3 and 12 months were 69 and 72%, respectively (Figure 1A). After multivariable LRMs predicting 3-month continence recovery, both BMI (OR 0.90, 95% CI 0.82–0.97, *p* value = 0.006) and BNS approach (OR 3.15, 95% CI 1.68–6.08, *p* value < 0.001) were independent predictors. Conversely, after multivariable LRMs predicting continence recovery at 12 months, only BNS (OR 3.51, 95% CI 1.87–6.67, *p* value < 0.001) was independent predictor (Table 2).

### Multivariable logistic regression models predicting continence at 3 and 12 months after RARP in prostate glands ≤ 40 mL

In RARP patients with prostate glands ≤ 40 mL, the rates of continence at 3 and 12 months were 61 and 73%, respectively (Figure 1B). After multivariable LRMs predicting 3 months continence recovery, only prostate-gland asymmetry (OR 0.33, 95% CI 0.13–0.83, *p* = *0.02*) was independent predictor (Table 3A). Conversely, after multivariable LRMs predicting continence recovery at 12 months, prostate-gland asymmetry (OR 0.31, 95% CI 0.10–0.90, *p* = 0.02), IPSS (OR 0.91, 95% CI 0.84–0.99, *p* = 0.02), as well as BNS approach (OR 5.10, 95% CI 1.67–16.73, *p* = *0.005*) were independent predictors (Table 3B).

### Multivariable logistic regression models predicting continence and early continence in prostate glands > 40 mL

In RARP patients with prostate glands ≤ 40 mL, the rates of continence at 3 and 12 months were 66 and 71%, respectively (Figure 1B). After multivariable LRMs predicting 3-month continence recovery, IPSS (OR 0.96, 95% CI 0.92–0.99, *p* = *0.03)* and BNS approach (OR 2.71, 95% CI 1.50–5.04, *p* = *0.001*) were independent predictors (Table 3A). Conversely, after multivariable LRMs predicting continence recovery at 12 months, BNS approach (OR 3.32, 95% CI 1.52–7.33, *p* = 0.003) was independent predictor. In both LRMs, the prostate-gland asymmetry did not show a statistically significant predictive value (Table 3B).

## Discussion

Post-operative UI remains one of the major concerns after RARP [5]. Historically, Ficarra et al., relied on meta-analysis methodology, including 51 studies, and showed an UI rate at 12 months ranging from 4 to 31%, with a mean value of 16% using a no pad definition [4]. Recently, several preoperative clinical and technical predictors of UI after RARP have been described, such as PV, prostate shape or bladder neck preservation surgical techniques as well as surgical urethral length preservation [9, 10, 14, 16, 20]. However, to the best of our knowledge no previous author investigated the role of prostate-gland asymmetry (defined as the presence of median lobe or side lobe dominance) in predicting continence after RARP. We addressed this void. Specifically, the aim of the current study is to test the impact of the prostate-gland asymmetry on 3- and 12-month continence recovery after RARP. Moreover, we also investigated the potential impact of prostate-gland asymmetry according to the PV, hypothesizing that effect of prostate-gland asymmetry on continence recovery might be different. We recorded several noteworthy observations.

First, we measured that more than half of our sample presented prostate-gland asymmetry. However, not clinically meaningful or statistically significant differences were observed in the proportions of favorable Q max, as well as unfavorable PVR. According to the PV, the vast majority of RARP patients exhibited a prostate gland > 40 mL, regardless the presence of prostate lobe asymmetry. Specifically, 46% of Group 1 and 28% of Group 2 RARP patients had a prostate gland ≤ 40 mL, while 54% of Group 1 and 72% of Group 2 RARP patients had a prostate size > 40 mL (*p* = 0.004). In conclusion, surgeons most often have to deal with patients characterized by irregular prostate shape, such as the presence of median lobe or large prostate size, making the surgery more difficult [21, 22]. This may be explained by the fact that the average age of RARP patients is usually over 65 years [23].

Second, from our analyses, it emerged that 69 and 72% of RARP patients in the overall population were continent within 3-month and 12-month post-surgery, respectively. Our results enlightened an improvement in continence rate within the 12 months after RARP. Moreover, this improvement was consistently with previous studies. For instance, Inoue et al. in their prospective report including 291 RARP patients, measured a continence rate at 3 and 12 months that ranged from 55.2% to 85%, respectively. Moreover, Nakane et al. recorded similar rates, analyzing a cohort of 73 patients who underwent RARP in a single center [24]. Indeed, from their findings, it emerged that urinary continence was restored in 53.4 and 84.9% of patients at 3 and 12 months after RARP, respectively [24]. Altogether, we performed two independent LRMs to investigate the potential predictors of 3- and 12-month continence recovery after RARP, in the overall population. From our analysis, it emerged that the bladder neck sparing approach was the strongest predictor of 3 (OR 3.15) and 1 month (OR 3.51) continence recovery (both *p* < 0.001). Moreover, also the BMI (OR 0.90) was an independent predictor of 3-month continence recovery (*p* = 0.006). Our results were consistent with Kim et al. findings on 452 RARP patients from a single center institution [25]. Indeed, they observed that lower BMI was an independent factor that predicted continence recovery within 3 months after RARP [25]. Moreover, we also agreed with Friedlander et al. findings [10]. The authors studied 791/1067 RARP patients undergoing bladder neck sparing vs non-sparing approaches [10]. They measured that BNS was associated with fewer urinary leak complications, shorter hospitalization and better post-prostatectomy continence [10]. Furthermore, Ippoliti et al. in meta-analyses of 39 randomized control trials, assessed the role of bladder neck sparing approach as potential predictor of continence [26]. However, from the above observations, the potential effect of prostate-gland asymmetry on continence recovery after RARP was not proved.

Third, we performed two independent subgroups analyses in RARP patients with PV  ≤ 40 as well as > 40 mL. Specifically, from our subgroup analyses on prostate glands ≤ 40, it emerged that 61% and 73% of RARP patients recovered continence at 3 and 12 months, respectively. Relying on LRM, the prostate-gland asymmetry negatively affected the achievement of both 3- and 12-month continence. Conversely, in subgroup analyses on prostate glands > 40 mL predicting continence at 3 and 12 months, the prostate lobe asymmetry failed to reach statistically significance. Our findings were partially in contrast to Martinez et al. analyses, on urinary functional outcomes of 220/663 RARP patients with enlarged mean lobes (ML) [27]. They observed that the presence of enlarged ML did not increase the risk of incontinence after RARP, instead it appeared that ML patients had greater improvements in post-surgical urinary functions [27]. However, they did not consider the presence of lobe side asymmetry and the PV that may have an effect in impairing the bladder neck reconstruction and thus the post-operative urinary functioning.

Our results strengthened the concept that the effect of prostate lobe asymmetry might depends on PV. Indeed, the magnitude of the above effect is much stronger in small prostates than their larger counterparts. The rationale behind these observations suggested that in larger prostates, regardless of the presence of prostate-gland asymmetry, the technical challenges might be greater. For instance, prostate glands > 40 mL may hinder the surgeon during the apex step of RARP, resulting in MUL sparing loss. Indeed, the continence recovery after RARP may be influenced in addition by the millimeters of MUL preserved during the procedure [16, 28]. For instance, Ragusa et al. observed within 100 PCa patients treated with laparoscopic RP that surgical urethral length preservation independently predicted urinary continence [16]. Furthermore, Kitamura et al. analyzing 320 patients who underwent RARP, found that a greater post-operative MUL facilitated the achievement of 3-month continence recovery after RARP [28]. Moreover, Paparel et al. in a retrospective report of 64 RARP patients, assessed that the MUL loss ratio (defined as the percent change between preoperative and post-operative MUL) was related to the recovery time after RP [29]. As a result, MUL loss and periurethral fibrosis impeded continence recovery by compromising the elasticity of external sphincter [29]. However, our hypothesis was speculative and needed further investigations in a prospective design study which weighted the MUL loss according to the PV.

The clinical implication of the current study may be applied as follows: first, the BNS approach, as previously defined, was independent predictor of continence and early continence despite the presence of prostate lobe asymmetry and PV. Second, in prostate glands ≤ 40, the prostate lobe asymmetry could impair the achievement of both 3- and 12-month continence, with a not negligible effect. This aspect should be integrated in the patients counseling and surgery planning to better definition of post-surgical outcomes in a tailored decision-making program.

Despite the novelty of our observation, the study presented several limitations. First, the retrospective nature of our study and the small sample size represented an inherent limitation. To overcome the above limitation a multicentric study could be designed. Second, the MRIs were performed in different Institutions, resulting in a bias related to image quality, cropping, and reproducibility, and invariably to a data loss. Third, we did not have detailed information on the type of NS procedure performed (if monoliteral or bilateral). However, this is common with all similar observational studies from single-tertiary referral centers. Moreover, some residual bias that may have affected our results cannot be excluded.

## Conclusions

The presence of prostate lobe asymmetry negatively affected the continence rates in prostate glands ≤ 40 mL at 3 and 12 months after RARP. These observations should be considered in the preoperative planning and counseling of RARP patients. However, future larger and multicenter studies are needed to prove or refuse our findings.

## Data Availability

The data and materials that support the findings of this study are available from the corresponding author upon reasonable request.
